# Necrotic thymoma with cardiac compression in a young lady

**DOI:** 10.1186/1749-8090-8-S1-P29

**Published:** 2013-09-11

**Authors:** E Likaj, A Kacani, S Dumani, D Hasi, A Alia, A Kenga, L Dibra, A Refatllari

**Affiliations:** 1 Cardiovascular Surgery Department, University Hospital Centre “Mother Theresa”, Tirana, Albania

## Background

Necrosis, infarction and cystic degeneration in thymoma are described mostly focally. Extensive cystic and necrotic degenerations are reported very rarely.

## Methods

A 30-year-old female patient presented with a history of three months complaining of interscapular chest pain and progressive dyspnea on exertion. Last two weeks she had nocturnal dyspnea and excessive chest pain. The patient had no clinical signs of myasthenia gravis. Radiological investigations revealed a mass lesion of 82x55x90 mm located in the anterior mediastinum without any calcifications or mediastinal extension. Echocardiography showed compression of the right ventricle with dilatation of the inferior vena cava.

## Results

The patient was operated on standard median sternotomy. During the operation, a well encapsulated large tumor tissue was observed. Macroscopically it was suggestive of thymoma with large areas of necrosis and caseous tissues. No infiltration of the pleura or pericardium was observed. The tumor was completely removed. The postoperative course was free of complications, except an inflammatory syndrome with subfebrile temperature ranging 37,5 to 38,0 for a period of 5 days. Histopathological examination confirmed the diagnosis of a type A thymoma with large areas of necrotic tissues and cystic degeneration. At control after four years no signs of recurrence of the disease are noted on computed tomography.

## Conclusions

Cardiac compression in thymoma is very rare pathology. Early diagnosis and surgical treatment is mandatory. Completeness of resection is a reliable prognostic factor.

